# Effect and Mechanism of Acid-Induced Soy Protein Isolate Gels as Influenced by Cellulose Nanocrystals and Microcrystalline Cellulose

**DOI:** 10.3390/foods11030461

**Published:** 2022-02-03

**Authors:** Xueqi Jin, Ruijing Qu, Yong Wang, Dong Li, Lijun Wang

**Affiliations:** 1 Beijing Key Laboratory of Functional Food from Plant Resources, College of Food Science and Nutritional Engineering, China Agricultural University, P.O. Box 50, 17 Qinghua Donglu, Beijing 100083, China; 13935285477@163.com (X.J.); qrj@cau.edu.cn (R.Q.); 2 School of Chemical Engineering, The University of New South Wales, Sydney, NSW 2052, Australia; benjaminwy@gmail.com; 3 Beijing Advanced Innovation Center for Food Nutrition and Human Health, National Energy R & D Center for Non-Food Biomass, College of Engineering, China Agricultural University, Beijing 100083, China; dongli@cau.edu.cn

**Keywords:** CNC, MCC, acid-induced soy protein isolate gelation, rheology, microstructure

## Abstract

The effects of cellulose nanocrystals (CNC) and microcrystalline cellulose (MCC) on the gel properties and microstructure of glucono-δ-lactone-induced soy protein isolate (SPI) gels were investigated. The water-holding capacity, gel strength, and viscoelastic modulus of CNC–SPI gels were positively associated with CNC concentration from 0 to 0.75% (*w*/*v*). In contrast, MCC–SPI gels exhibited decreased water-holding capacity, gel strength, and viscoelastic modulus. All composite gels displayed high frequency dependence and the typical type *I* (strain thinning) network behavior. Changes in viscoelasticity under large strain were correlated with differences in the microstructure of SPI composite gels. Scanning electron microscopy (SEM) and confocal laser scanning microscopy (CLSM) showed that CNC were more evenly and steadily distributed in the protein matrix and formed a compact network structure. In contrast, MCC–SPI gels exhibited a discontinued and rough gel network with some large aggregates and pores, in which MCC was randomly entrapped. Fourier transform infrared spectroscopy (FTIR) and molecular forces results revealed that no new chemical bonds were formed in the gelation process and that the disulfide bond was of crucial importance in the gel system. With the addition of CNC, electrostatic interactions, hydrophobic interactions, and hydrogen bonds in the SPI gel network were significantly strengthened. However, the incorporation of MCC might obstruct the connection of the protein network. It is concluded that both cellulose type and concentration affect gelling properties.

## 1. Introduction

Soy protein isolate (SPI), which mainly contains globulins glycinin (11S) and β-conglycinin (7S), has been extensively used in the food field due to its high nutritive values and functional properties [1]. In addition, as a plant-derived protein, SPI has attracted widespread attention due to its low cost, low pollution, low toxicity, and renewable property compared with animal-based proteins [2,3]. The formation of gel is regarded as one of the most important functions. Acid-induced soy protein gel can be achieved by preheating the protein solution to aggregates and then lowering the pH toward the isoelectric point by adding glucono-δ-lactone (GDL) to reduce intermolecular repulsion [4]. However, pure protein gels are sensitive to environmental conditions and have poor stability, which significantly limit their application [5]. 

It has been demonstrated that polysaccharides can alter the functional properties of protein gels, resulting in the formation of resultant composite gels with different microstructural, mechanical, and sensory properties [6]. The protein–polysaccharide interactions can be either adverse or beneficial to the formation of gels depending on their nature, addition amount, and ratio as well as the gel conditions [7]. Thus, it is vital to systematically investigate protein–polysaccharide interactions in order to design and develop the desired texture and expand the applications of gels.

Cellulose nanocrystals (CNC), one of the nanocelluloses with rod structure, are typically obtained by sulfuric acid hydrolysis and have attracted extensive attention due to their unique physicochemical characteristics [8]. The high aspect ratio and Young’s modulus, together with large specific surface area, richness in hydroxyl groups, negligible toxicity, and excellent biodegradability and biocompatibility, offer extensive application prospects for CNC in many fields [9]. Currently, the potential applications of CNC in the food industry have been deeply studied, such as to stabilize the emulsion, act as thickeners and flavor carriers, modify the digestion protein, and improve food packaging [10,11,12]. Microcrystalline cellulose (MCC), an insoluble hydrophilic cellulose derivate, is a white crystalline powder that has been approved for use as food additive [13]. It is used as a stabilizer, sorbent, and filler in food products and as a potential fat replacer [14,15].

In recent years, several studies have shown that insoluble cellulose significantly affects protein gelation. Zhuang et al. [16] demonstrated that sugarcane dietary fiber (420 and 177 μm) was trapped within the myofibrillar protein gels and improved gel functionality by changing the water distribution. Xiao et al. [17] revealed that the addition of CNC remarkably improved heat-induced whey protein isolate gels and exhibited better WHC, thermal stability, and textural and rheological properties. They also contributed to forming a uniform and dense gel structure. However, Ahmadi et al. [14] reported that both micro- and nanocrystalline cellulose decreased the viscoelastic modulus of whey protein gels and led to inhomogeneous rough networks. Ullah et al. [18] found that nanosized dietary fibers (370 nm) were distributed into tofu gel more uniformly and steadily than microsized dietary fibers (110 μm) but obstructed the gelling properties more obviously. 

In previous studies, researchers have focused more on heat-induced protein gels. To our knowledge, the acid-induced gelation mechanism for SPI gels loaded with cellulose crystalline particles is not entirely understood. It will be very interesting to explore the characteristics of MCC- and CNC-loaded protein gels, which could act as delivery systems for bioactive compounds. Furthermore, cellulose crystals can act as functional ingredients due to their nutritional properties and health benefits [11]. In this study, the effect of different sizes (nanometer and micrometer) and concentrations of cellulose crystals on SPI gels were elucidated. Small amplitude shear tests (SAOS) and large amplitude shear tests (LAOS) were used to analyze the rheological properties. Moreover, the water-holding capacity and microstructure of acid-induced SPI gels, along with their molecular forces, were also researched to propose a potential forming mechanism that can be helpful in expanding the application of cellulose in the food industry.

## 2. Materials and Methods

### 2.1. Materials

SPI (SD-100) (protein content ≥90%, dry basis, PI = 4.5) was supplied by Pine Agritech Limited (Shandong, China). CNC and MCC were both acquired from North Century Cellulose Material Co., Ltd. (Jiangsu, China) and prepared by sulfuric acid hydrolysis from cotton pulp. The zeta potential value of CNC was −45.7 ± 2.3 mV, and the length and diameter of CNC were 100–500 and 10–50 nm, respectively. The zeta potential value of MCC (~20 µm) was −10.3 ± 1.9 mV. GDL and fluorescent brightener 28 were obtained from Shanghai Macklin Biochemical Co., Ltd. (Shanghai, China). Rhodamine B was provided by Beijing Yinghai fine chemical industry (Beijing, China). All other chemicals used in this study were of analytical grade.

### 2.2. Preparation of Stock Solutions and Hydrogels

The gel samples were prepared using the methods reported by Bi et al. and Yan et al. [19,20] with a minor modification. Briefly, SPI power (8%, *w*/*v*) was dispersed in deionized water and stirred for 4 h at room temperature. Then, the solution was stored overnight at 4 °C for complete hydration, after which it was subjected to heat treatment at 90 °C for 30 min in a water bath, followed by rapid cooling in tap water. The pH of the SPI dispersion was adjusted to 7.0. Suspensions of CNC or MCC (2%, *w*/*v*) were prepared by dispersing powders in deionized water at room temperature by stirring for 1 h and then keeping it at 4 °C overnight.

At room temperature, the SPI was mixed with CNC by magnetic stirring for 20 min. All of the mixtures had the same final SPI concentration (5%, *w*/*v*), and the concentrations of CNC were 0, 0.25, 0.5, and 0.75% (*w*/*v*). After the addition of GDL (2%, *w*/*v*), the dispersions were immediately transferred to a 40 °C water bath and then incubated for 30 min to form CNC–SPI mixed gels, followed by rapid cooling in an ice-water bath. MCC–SPI mixed gels (5% SPI; 0, 0.25, 0.5, and 0.75% MCC) were obtained following the same method. All gels were stored at 4 °C overnight before further analysis.

### 2.3. Water-Holding Capacity (WHC) and Gel Strength

The WHC of SPI gels was determined based on the methods reported by Campbell et al. [21] with some modifications. Gel samples of around 5 g were centrifuged at 7000× *g* for 10 min at 4 °C. The WHC (%) was defined as the percentage of gel weight after centrifugation relative to its initial weight.

A texture analyzer (TA-XT plus, Stable Micro System Co., UK) equipped with a P/0.5R cylindrical probe was used for measuring the gel strength of SPI gel samples. First, 30 mL gel samples were formed in 50 mL texture cups. The gels were then equilibrated at room temperature for 30 min before analysis. The gels were penetrated to a depth of 5 mm at a speed of 1 mm/s, and the trigger force was 5 g.

### 2.4. Rheology Tests

Rheology measurements were performed using an AR2000ex rheometer (TA, Crawley, UK) with an aluminum parallel plate geometry (40 mm diameter, 1000 μm gap). The acid-induced SPI gels were formed in situ on a Peltier plate. The dispersions mixed with GDL were immediately transferred between the plates of the rheometer and then surrounded by dimethyl silicone oil to minimize water evaporation during measurements.

#### 2.4.1. Gel Formation

The samples were rapidly heated to 40 °C and kept for 30 min. During the dynamic oscillatory time sweep tests, the storage moduli (G′) was monitored as a function of time at a frequency of 1 Hz and a strain of 0.5% (within linear viscoelastic region). A kinetics model was used to characterize the relationship between G′ and time [19].

(1) $$G^{\prime }\left(t\right){=G}_{\infty }^{\prime }\left({1\;-\;e}^{-k\left({t\;-\;t}_{g}\right)}\right)$$

where $G_{\infty }^{\prime }$ indicates the estimated maximum G′ value of gel sample (Pa), k stands for the gelation rate constant (s^−1^), and t_g_ is the time when gelling starts (s).

#### 2.4.2. Frequency Sweep Test 

Once the gelling process was complete, the sample was rapidly cooled to 20 °C for 5 min to prevent s decline in pH values, after which it was subjected to a frequency sweep ranging from 0.1 to 10 Hz at a strain of 0.5%. The storage and loss moduli (G′ and G″) were monitored and recorded. A power law model was used to characterize the frequency dependencies of G′ and G″ [22].

(2) $$G^{\prime }=K^{\prime }\;\cdot {\;\omega }^{n\prime }$$

(3) $$G\prime \prime =K\prime \prime \;\cdot {\;\omega }^{n\prime \prime }$$

where K′ and K″ represent power law constants (Pa/s^n^), and n′ and n″ are frequency indices (dimensionless).

#### 2.4.3. Large Amplitude Oscillatory Shear (LAOS) Test

Large amplitude oscillatory shear (LAOS) test was performed at 20 °C with strain sweep. The strain applied ranged from 0.1 to 100% at 1 Hz. Raw data of the oscillation waveform was collected, and the MITlaos program (MITlaos beta) was used to analyze Lissajous curves [23,24].

### 2.5. Scanning Electron Microscope (SEM)

The microstructure of SPI gels was observed using the methods reported by Xiao et al. [25] with a minor modification. Gel samples were cut into cubes (2 mm^3^), immersed in 2.5% glutaraldehyde at 4 °C for 6 h, and rinsed with phosphate buffer (0.1 mol/L, pH 7.0) three times, followed by dehydration using ethanol solutions with gradient concentrations (30, 50, 70, 80, 90, and 100%). After freeze-drying, gels were coated with gold and observed by SEM (SU3500, HITACHI, Japan) at 10 kV. Images were taken at 3000× magnification.

### 2.6. Confocal Laser Scanning Microscopic (CLSM)

A Leica CLSM (TCS SP5 Ⅱ, Leica Microsystems CMS GmbH, Mannheim, Germany) equipped with an inverted microscope (Leica DMI6000, Wetzlar, Germany) was used to investigate the microstructure of SPI gels according to a previous study [19]. Rhodamine B (0.1%, *w*/*v*) and fluorescent brightener 28 (0.1%, *w*/*v*) were used for staining proteins and CNC/MCC, respectively [11]. Before gelation, samples were stained with fluorescent dyes in a volume ratio of 100:1:1 and mixed well. The mixture was added onto a concave slide and sealed, followed by gelling at 40 °C for 30 min. Rhodamine B and fluorescent brightener 28 were excited at 543 and 405 nm, respectively, and the emission wavelengths were collected at 580 and 441 nm, respectively. Images were captured at 100× magnification with a scale of 1024 × 1024 pixels.

### 2.7. Fourier Transform Infrared Spectroscopy (FTIR)

FTIR spectra of CNC, MCC, and SPI gels were recorded using a PerkinElmer infrared spectrometer (Spectrum 100, Perkin-Elmer Co., Waltham, MA, USA). The freeze-dried sample (2 mg) was thoroughly milled with KBr power (200 mg) and then compressed into a thin film. Measurements were performed at room temperature and subjected to 32 scans in the scanning range of 4000–400 cm^−1^ with a spectral resolution of 4 cm^−1^.

### 2.8. Measurement of Gel Solubility 

In order to explore the molecular forces of SPI gel, CNC–SPI gels, and MCC–SPI gels, gel solubility in different solvents was determined according to a previous study with some modifications [26,27]. The solvents were as follows: deionized water (S1), 0.086 mol/L Tris-0.09 mol/L glycine-4 mmol/L Na_2_EDTA (pH 8.0) (S2), S2 containing 0.5% SDS (S3), S3 containing 8 mol/L urea (S4), and S4 containing 2% β-mercaptoethanol (S5). Briefly, 0.4 g gel sample was thoroughly mixed with 10 mL of each solvent on a Vortex-Genie 2 mixer (Scientific Industries, INC., New York, NY, USA) for 2 min. Then, the mixtures were incubated at 25 °C for 20 min in a shaking water bath followed by centrifugation at 5000× *g* for 15 min. The concentration of protein in the supernatant was measured using the BSA standard. Protein solubility was expressed as the ratio of the supernatant protein content to total protein content.

### 2.9. Statistical Analysis 

All experiments were carried out in at least triplicate and are represented as mean ± standard deviation. Statistical analysis was conducted by SPSS software (SPSS 20.0; IBM SPSS Statistics, Chicago, IL, USA) and MATLAB software (MATLAB R2012a, Natick, MA, USA). Differences were considered statistically significant (*p* < 0.05) using the one-way analysis of variance (ANOVA) with Duncan’s test.

## 3. Results

### 3.1. WHC and Gel Strength

**Figure 1:**
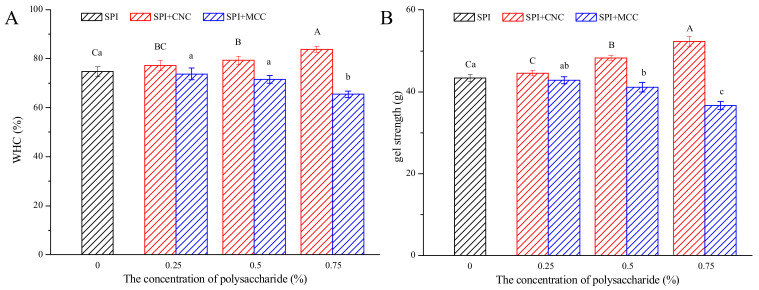
Water-holding capacity (**A**) and gel strength (**B**) of SPI gels containing different concentrations of CNC or MCC. Different upper case letters indicate significant differences among SPI gels containing different concentrations of CNC. Different lower case letters indicate significant differences among SPI gels containing different concentrations of MCC (*p* < 0.05).

Water-holding capacity (WHC) is one of the important parameters of gel systems. It is closely related to the interactions between water and other components in the gel as well as the network structure [22]. As shown in Figure 1A, WHC of SPI gels containing CNC and MCC showed a contrary trend with increasing cellulose concentration. The WHC values of the CNC–SPI gels increased significantly with increasing CNC concentration. Addition of MCC lower than 0.5% did not affect WHC significantly, while higher MCC concentration (0.75%) caused obvious reduction in WHC of about 9% (*p* < 0.05).

In a composite gel system, water molecules may bind to functional groups of polysaccharides and proteins or get held in small meshes and pores of the gel network [28]. CNC are highly hydroxylated and contain abundant hydrophilic groups [29]. Hence, the increased number of hydrogen bonds might be one reason for the increase in WHC. Moreover, improvement in the WHC of CNC–SPI gels may be attributed to stronger intermolecular interaction in the gel system and the formation of a firm and stable network [25]. The results were in agreement with the study by Xiao et al. [17], who found that CNC could enhance WHC, gel strength, and thermal stability of heat-induced whey protein isolate gels and result in the formation of a well-structured gel network. However, MCC had an adverse effect on WHC of SPI gels. A similar trend was observed by López et al. [30], who found that sodium caseinate acid-induced gels containing espina corona gum presented lower WHC. The interaction between protein and MCC was limited due to its lower surface charge, which could make them more susceptible to rearrangements and induce the formation of locally denser clusters and larger pores [31]. Large pores have lower potency to immobilize water, allowing water to flow out easily under centrifugal forces [32]. Moreover, MCC tends to aggregate in high concentrations, which might obstruct protein–protein interactions. The formation of phase-separated structure might also have accounted for the decrease in WHC [33].

As shown in Figure 1B, incorporation of CNC and MCC significantly influenced gel strength. The results followed a similar trend to WHC, with CNC increasing the gel strength in a concentration-dependent manner and the addition of MCC decreasing the gel strength. It is generally accepted that gel strength is related to the arrangement of the protein network [17,22]. The incorporation of CNC and MCC might have affected the protein–protein interactions and protein aggregation, thus exhibiting different texture parameters. An increase in gel strength with increasing CNC concentration suggests the formation of a homogeneous and cross-linked protein network. Insoluble large particles affect protein chain association, reducing protein–protein interaction [18]. Thus, the decrease in gel strength of MCC–SPI gels might be attributed to the decrease in protein–protein interaction and an unstable network structure. The results were consistent with the changes in interaction forces. In brief, the results of the WHC and gel strength prove that the addition of cellulose is an effective way to tune the characteristics of SPI gels. 

### 3.2. Rheological Tests 

#### 3.2.1. Gel Formation 

**Figure 2:**
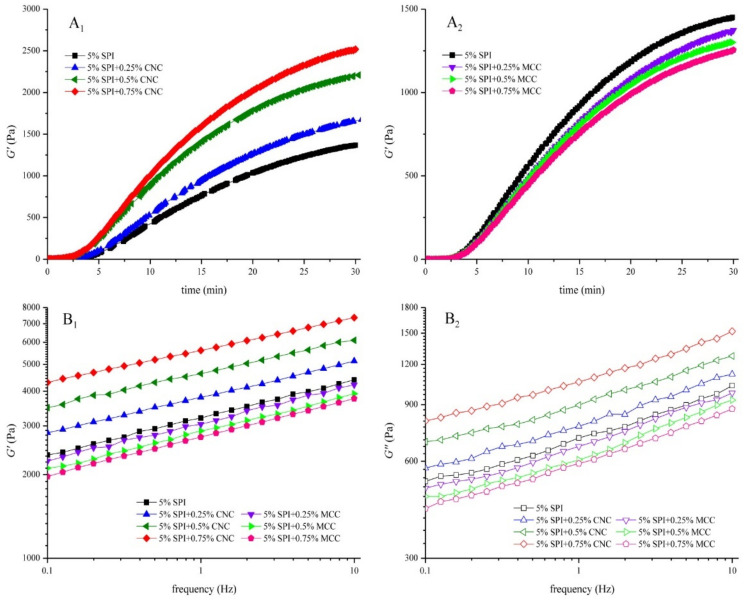
Linear rheology properties: time dependence of storage modulus G′ for CNC–SPI gels (**A_1_**) and MCC–SPI gels (**A_2_**); changes in frequency dependence of storage modulus G′ (**B_1_**) and loss modulus G′′ (**B_2_**).

The elastic modulus (G′) depends on the number of effective elastic junctions between strands [34]. Figure 2A_1_,A_2_ shows G′ development for CNC–SPI gels and MCC–SPI gels during their gelation processes, respectively. The tendency of G′ with time was similar. GDL hydrolyzed progressively, releasing protons to neutralize the negative charges on SPI as the pH value decreased. When the formation of SPI gel started, G′ increased sharply and then reached a maximum value. A similar gelation trend was observed by others [35].

As summarized in Table 1, the kinetics model was used to fit the time sweep data using Equation (1) (*R*^2^ > 0.99). CNC exhibited noticeable increase in G′ of SPI gels, indicating a strong interaction between protein molecules and cellulose chains. $G_{\infty }^{\prime }$ increased and the gelation rate constant k and the gelation time t_g_ decreased significantly when the concentration of CNC increased (*p* < 0.05). The rod structure of CNC leads to great resistance [36]. The incorporation of CNC may strengthen the protein–protein interactions with increased relative density of protein aggregates, thus accelerating the early stage of gel formation [34,37]. Moreover, CNC could act as a thickening agent and might form hydrogen bonds with SPI [29].

However, in the case of MCC–SPI, $G_{\infty }^{\prime }$ decreased while the gelation time t_g_ showed no significant changes with increasing concentration of MCC (*p* < 0.05). G′ is closely related to the network structure and bond strength of the gel [38]. The incorporation of MCC might obstruct the connection of the protein network, thus reducing the number of effective strands and leading to a rougher network [18]. Similar findings were reported in other protein–polysaccharide gel systems [39].

#### 3.2.2. Frequency Dependence 

The frequency sweep plots of SPI gels are shown in Figure 2B_1_ and Figure 2B_2_. The storage and loss moduli for all samples went up with the increase in frequency, which might be due to the acceleration of molecular mobility at high frequencies [40]. The strong frequency dependence indicates that the network was mainly formed by noncovalent physical crosslinks [41]. G′ was far larger than G”, suggesting that all gels tended to be more elastic or solid-like. Consistent with the time sweep results, G′ and G″ values of CNC–SPI gels increased with CNC concentration. In contrast, G′ and G″ values showed a negative correlation with the concentration of MCC.

As shown in Table 2, the power law model fit well with the experimental data (*R*^2^ > 0.99). In the case of CNC–SPI gels, the values of K′ and K″ increased significantly with increased CNC concentration (*p* < 0.05), indicating that the incorporation of CNC enhanced the viscoelasticity. The values of n′ and n″ decreased after the addition of CNC (*p* < 0.05), suggesting that the sensitivity of the gels to the frequency was reduced. This might be due to the crystalline structure of CNC limiting polymer mobility [36]. The values of K′ and K″ decreased significantly after the addition of MCC, while n′ increased and n″ saw no significant difference (*p* < 0.05). This indicates that incorporation of MCC weakened the viscoelasticity of SPI gel, increased the elastic frequency dependence, and exerted no significant effect on the viscous frequency dependence.

#### 3.2.3. Nonlinear Rheology Properties 

**Figure 3:**
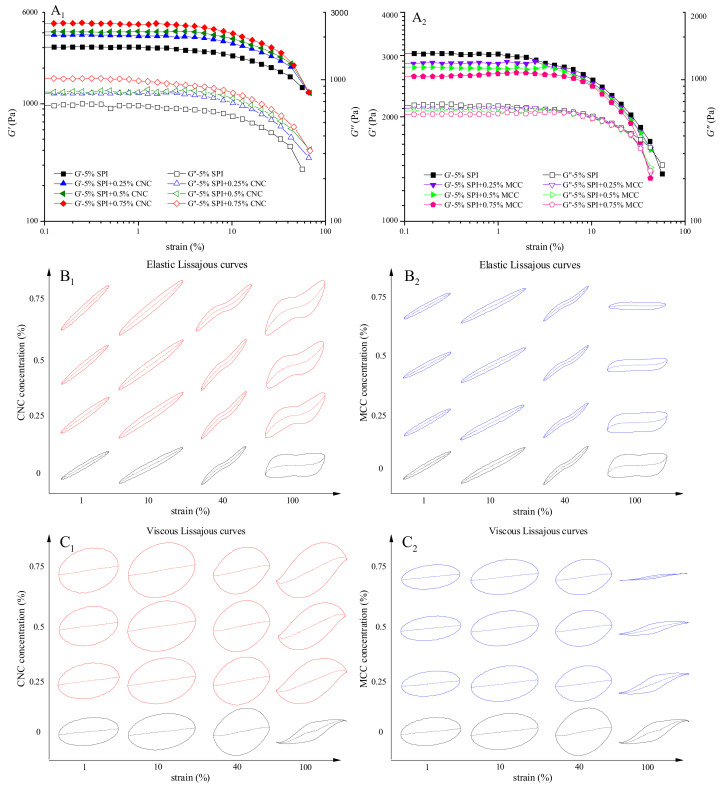
Nonlinear rheology properties: large amplitude oscillatory shear behavior of CNC–SPI gels (**A_1_**) and MCC–SPI gels (**A_2_**); elastic Lissajous curve of CNC–SPI gels (**B_1_**) and MCC–SPI gels (**B_2_**), and viscous Lissajous curves of CNC–SPI gels (**C_1_**) and MCC–SPI gels (**C_2_**). The black line is SPI gel, red line is CNC–SPI gels, and blue line is MCC–SPI gels in Lissajous curves. The coordinate axes of the same column in Lissajous curves are the same, and the solid and dashed lines represent the total stress and the decomposed stress, respectively.

The nonlinear rheology properties of gels reflect the sensory quality and mechanical properties related to food processing and contain valuable information about the microstructure [42]. The LAOS behaviors of SPI gels are shown in Figure 3A_1_,A_2_. G′ and G″ values of all gel samples remained constant regardless of strain amplitude at the linear viscoelastic region and then decreased dramatically with further increase in strain amplitude. This can be classified as type *I* gels (strain thinning). The creation rate parameter was negative (a < 0) and the decomposition rate parameter was positive (b > 0), indicating that the SPI gels were broken down easily and that the protein molecules had little chance to reestablish the network [24]. The number and types of protein–protein bonds, the aggregated size and shape during the “preaggregation” stage, and the gel network all influence the nonlinear viscoelastic behavior of gels [22,43]. Increasing CNC concentration increased both G′ and G″, which might be due to the favorable interaction between CNC and protein molecules [17,44]. The values of G′ and G″ decreased after the addition of MCC. It is noted that the decrease in viscoelastic moduli of MCC–SPI gels was slower than that of SPI gel under medium strain, which might be attributed to the energy dissipation caused by disruption of extensive agglomerates [45].

Lissajous curves show the nonlinear behaviors of gels, with the shape and area of a Lissajous curve implying the viscoelastic response. The total stress is decomposed into elastic stress and viscous stress. The elastic Lissajous curves of SPI gels are depicted in Figure 3B_1_,B_2_, and the viscous Lissajous curves are presented in Figure 3C_1_,C_2_. As the systems entered the nonlinear region, the shape of the elastic Lissajous loops deformed from an ellipse to a rectangle and the strain rate curves changed from straight lines to distorted curves, indicating that the decrease in elasticity was faster than viscosity [46]. The area of the Lissajous loops expanded obviously with the addition of CNC, demonstrating that the viscoelasticity of the gels increased [47]. This might be due to the strong resistance to permanent deformation by the formation of a well-structured gel network [48]. The incorporation of MCC might have formed a rougher gel network. The formation of fractures around the weakest junctions and existing pores would thus become more probable under large strain, which might account for the decrease in area of the Lissajous loops under large strain [49].

### 3.3. Morphology and Microstructure 

#### 3.3.1. SEM 

**Figure 4:**
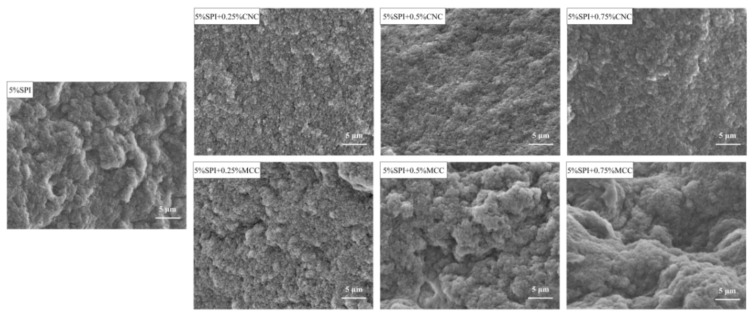
SEM images of SPI gels containing different concentrations of CNC or MCC.

The microstructures of SPI gels with or without different additions of CNC and MCC are presented in Figure 4. The control SPI gel showed a rough and disordered network with some large pores, indicating that the protein molecules were not well connected. The addition of CNC exhibited a smoother surface with more compact pores, facilitating the formation of homogeneous and dense networks. However, the gel network became disordered and fragmented with addition of MCC as evidenced by irregular aggregates. This might be responsible for the decreased WHC [31]. The large specific surface area of CNC was instrumental in binding the water molecules. The water might have migrated from the protein matrixes, resulting in “concentrated” SPI and thereby enhancing the gel network [16]. In contrast, MCC might have served as a passive filler that was not tightly embedded within the gel network, thus leading to decreased gel quality.

#### 3.3.2. CLSM

**Figure 5:**
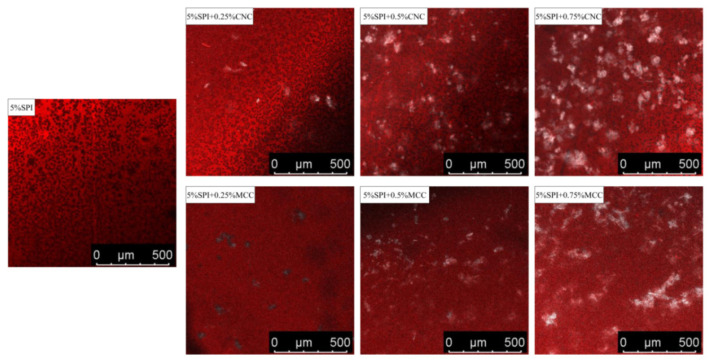
CLSM images of SPI gels containing different concentrations of CNC or MCC.

Figure 5 shows the CLSM images of SPI gel systems with or without CNC and MCC. Protein stained with rhodamine B appears as red, while CNC or MCC stained with fluorescent brightener 28 appear as white. The presence of CNC and MCC affected the distribution and size of the cavities. The pure SPI gel showed a heterogeneous network with different pore sizes. When CNC content increased, the protein continuous phase exhibited more compact and uniform structures, and the density of protein aggregates increased. The agglomerated CNC served as “active” fillers that were independently distributed in the gels [17]. However, MCC–SPI gels displayed inhomogeneous and loose microstructures with some large pores, where MCC was irregularly distributed in the gel matrix and tended to aggregate. Consistent with a previous study [18], CNC were distributed more uniformly into SPI gels compared with MCC, which might be due to the nanosize effect. The microstructure of protein–polysaccharide gel systems is determined by the competition between “gelation” and “phase separation” [4]. These CLSM images suggest that thermodynamic incompatibility occurred more dominantly between MCC and SPI, while “gelation” was more significant in CNC–SPI gels.

### 3.4. Molecular Forces 

**Figure 6:**
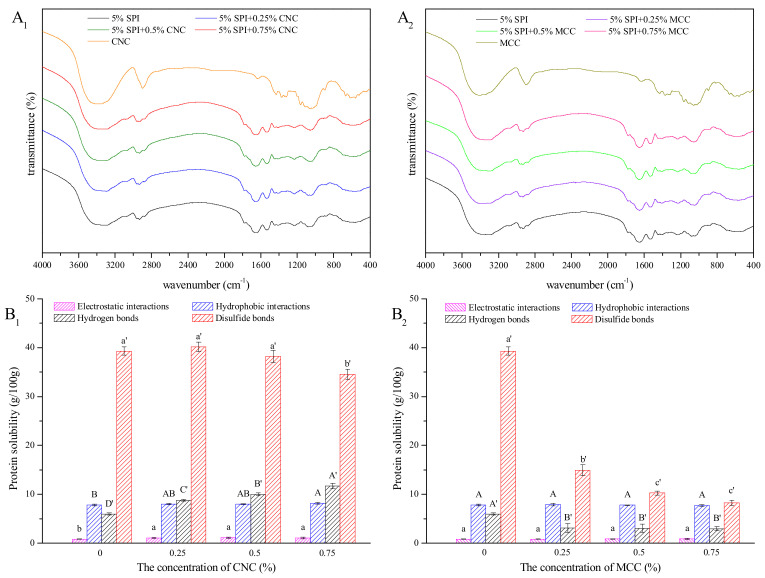
Molecular forces. FTIR spectra of CNC and SPI gels containing different concentrations of CNC (**A_1_**), MCC and SPI gels containing different concentrations of MCC (**A_2_**), and chemical interaction forces of CNC–SPI gels (**B_1_**) and MCC–SPI gels (**B_2_**). Different letters indicate significant differences (*p* < 0.05).

The FTIR spectra of CNC, MCC, and SPI gels are shown in Figure 6. The broad band at 3000–3700 cm^−1^ can be attributed to the O–H and N–H groups, reflecting more abundant hydroxyl groups on the surface of CNC compared to MCC [14]. This study found no clear changes in SPI gel spectra, indicating that no new chemical bonds formed in the gelation process. These results are consistent with previous studies showing physical entanglement and noncovalent interactions are the predominant driving forces between cellulose molecules and protein molecules [14,17]. The interaction mechanisms of the composite gels still need further investigated. 

The protein solubility of SPI gels with or without CNC or MCC in five types of solvents were determined to explore the contribution of noncovalent and covalent bonds. The differences between two adjacent solvents represent electrostatic interaction, hydrophobic interaction, hydrogen bond, and disulfide bond [27]. As shown in Figure 6, hydrophobic interaction, hydrogen bond, and disulfide bond played a major role in the formation of SPI gels, where disulfide bond is of crucial importance. The number and accessibility of disulfide bonds are increased by heat treatment of SPI dispersion and GDL-induced gels pre-solutions [21]. Moreover, soluble SPI aggregates produced from high SPI concentration exhibit high contents of free sulfhydryl groups [35]. These might have accounted for the high disulfide bonds in the gels. Hydrophobic interaction contributed less in this study compared to a previous study [27], which might be due to the formation of larger aggregates after adjusting the pH with acid [49]. Furthermore, the unfolding of protein molecules with high SPI concentration was slightly hindered, and the exposed hydrophobic groups tended to re-encapsulated due to the association between thermal aggregates [35]. With the addition of CNC, electrostatic interactions, hydrophobic interactions, and hydrogen bonds in SPI gel networks were significantly strengthened (*p* < 0.05), further verifying that CNC serve as a dehydrating agent to concentrated SPI solution. Moreover, the incorporation of CNC gives rise to more hydrogen bonds participating in the formation of the gel network. However, as the concentration of CNC increased to 0.75%, disulfide bonds in SPI gel networks saw a weak decline (*p* < 0.05), which might be due to the steric effect of the self-agglomerated CNC [50]. Electrostatic attractive forces and hydrogen bonding existed between CNC and SPI, while the interaction between MCC and SPI was limited. For MCC–SPI gels, hydrogen bonds and disulfide bonds were significantly decreased compared to pure SPI gel (*p* < 0.05). The incorporation of MCC might obstruct the connection of the protein network and have adverse effect on the accessibility of disulfide bonds, which further explains the decreased moduli and WHC [21,51].

### 3.5. Schematic Mechanism 

**Figure 7:**
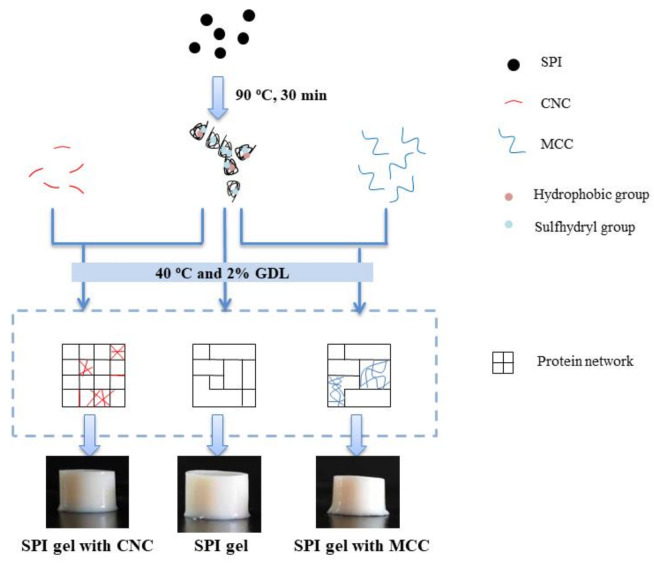
The suggested mechanism for the effects of CNC and MCC on acid-induced gelation of SPI.

Based on the above results, the potential mechanism for GDL-induced gelation of SPI with or without CNC or MCC is elucidated in Figure 7. The pure SPI gel exhibited a disordered and porous structure. 

The rod-shaped CNC have large specific surface area, good biocompatibility, and nanosize effect, which is highly hydroxylated and endowed with unique physicochemical properties. CNC might compete with proteins for water molecules and change the water distribution, leading to “concentrated” SPI and enhancing the gel network. The nanoscale CNC were more uniformly distributed in SPI gels, were steady, and tended to agglomerate. They might have acted as an active filler and contributed to the physical entanglement and hydrogen bonds. As the concentration of CNC increased, electrostatic interactions, hydrophobic interactions, and hydrogen bonds in the SPI gel network were strengthened, but higher CNC concentration (0.75%) caused a weak reduction in disulfide bonds. In any case, the incorporation of CNC promoted the formation of a compact, regular, and firm GDL-induced SPI gel network with improvement in functional properties.

In contrast, the micrometer-scale MCC with fewer surface charges was irregularly and randomly distributed in SPI gels, which acted as a passive fill within the protein and influenced the gel network integrity and continuity. Moreover, after the incorporation of MCC, hydrogen bonds and disulfide bonds in the gels were significantly decreased. Thermodynamic incompatibility seemed to occur more dominantly and before protein gelation, which promoted the formation of microphase separation structures, resulting in rough, disordered, and uneven network structures that corresponded to low WHC, gel strength, and viscoelastic moduli.

## 4. Conclusions

In this study, the effects of addition of CNC and MCC, which could act as potential functional ingredients, on acid-induced SPI gels were investigated. The significant improvement in WHC, gel strength, and rheological properties of CNC–SPI gels indicate that the gel network structures were strengthened. Naturally sourced CNC can be used as a potential gel modifier, which plays a key role in forming CNC–SPI gels by changing the water distribution and constructing a more compact and homogeneous network structure. In contrast, the incorporation of MCC decreased WHC, gel strength, and G′ and G″ value of gels and impeded the protein gelation process, resulting in the formation of a rougher network with some large aggregates and pores. Both the type and concentration of cellulose significantly affected the formation of acid-induced SPI gels. Unexpectedly, CNC and MCC had the opposite effect when introduced into SPI gels, with the strengthening effect of CNC proving much greater than the weakening effect of MCC. These findings provide a preliminary understanding of the interaction between protein molecules and cellulose chains, which can be applied to tune the characteristics of SPI gels, develop novel food products, and expand the potential application of cellulose in the food industry. Further work should focus on the competition between “phase separation” and “protein network formation” of composite gels and the design of delivery systems for bioactive compounds.

## Figures and Tables

**Table 1:** The parameter fitting results of the kinetics model for CNC–SPI gels and MCC–SPI gels.

Sample	$G^{\prime }\left(t\right){=G}_{\infty }^{\prime }\;\left({1\;-\;e}^{-k\left({t\;-\;t}_{g}\right)}\right)$			*R* ^2^
(*w*/*v*)	$G_{\infty }^{\prime }\;(\mathrm{Pa})$	k (s^−1^)	t_g_ (s)	
5%SPI	2389 ± 26 ^Da^	0.00071 ± 0.00001^Aa^	213.93 ± 5.50 ^Aa^	0.995
5%SPI + 0.25%CNC	2930 ± 16 ^C^	0.00057 ± 0.00005 ^BC^	205.13 ± 8.15 ^A^	0.995
5%SPI + 0.50%CNC	3470 ± 65 ^B^	0.00065 ± 0.00010 ^AB^	135.43 ± 21.06 ^B^	0.992
5%SPI + 0.75%CNC	4598 ± 47 ^A^	0.00051 ± 0.00008 ^C^	129.97 ± 8.69 ^B^	0.990
5%SPI + 0.25%MCC	2193 ± 10 ^b^	0.00065 ± 0.00001 ^b^	203.70 ± 8.01 ^a^	0.995
5%SPI + 0.50%MCC	2029 ± 64 ^c^	0.00070 ± 0.00003 ^a^	199.03 ± 2.70 ^a^	0.995
5%SPI + 0.75%MCC	2004 ± 78 ^c^	0.00065 ± 0.00003 ^b^	208.97 ± 11.27 ^a^	0.995

Values are the mean values ± standard deviation (*n* = 3). Different upper case letters superscripted indicate significant differences among SPI gels containing different concentrations of CNC (*p* < 0.05). Different lower case letters superscripted indicate significant differences among SPI gels containing different concentrations of MCC (*p* < 0.05).

**Table 2:** The parameter fitting results of power law model for CNC–SPI gels and MCC–SPI gels.

Sample	$G^{\prime }=K^{\prime }\;\cdot {\;\omega }^{n^{\prime }}$			$G^{\prime \prime }=K^{\prime \prime }\;\cdot {\;\omega }^{n^{\prime \prime }}$		
(*w*/*v*)	$K^{\prime }$	$n^{\prime }$	*R* ^2^	$K^{\prime \prime }$	$n^{\prime \prime }$	*R* ^2^
5%SPI	2541.7 ± 39.2 ^Da^	0.1339 ± 0.0017 ^A^^a^	0.999	530.8 ± 12.9 ^Da^	0.1544 ± 0.0011 ^Aa^	0.995
5%SPI + 0.25%CNC	3015.6 ± 13.8 ^C^	0.1278 ± 0.0008 ^B^	0.999	597.3 ± 4.8 ^C^	0.1487 ± 0.0027 ^A^	0.996
5%SPI + 0.50%CNC	3604.5 ± 51.5 ^B^	0.1230 ± 0.0004 ^C^	0.999	701.1 ± 17.7 ^B^	0.1382 ± 0.0052 ^B^	0.992
5%SPI + 0.75%CNC	4515.0 ± 28.2 ^A^	0.1160 ± 0.0002 ^D^	0.999	820.3 ± 9.6 ^A^	0.1392 ± 0.0009 ^B^	0.994
5%SPI + 0.25%MCC	2361.9 ± 82.8 ^b^	0.1364 ± 0.0010 ^b^	0.999	505.2 ± 12.6 ^a^	0.1545 ± 0.0076 ^a^	0.994
5%SPI + 0.50%MCC	2197.9 ± 46.3 ^c^	0.1367 ± 0.0013 ^b^	0.999	467.4 ± 11.6 ^b^	0.1561 ± 0.0057 ^a^	0.996
5%SPI + 0.75%MCC	2067.0 ± 124.8 ^c^	0.1383 ± 0.0013 ^b^	0.999	438.8 ± 28.1 ^b^	0.1562 ± 0.0005 ^a^	0.995

Values are the mean values ± standard deviation (*n* = 3). Different upper case letters superscripted indicate significant differences among SPI gels containing different concentrations of CNC (*p* < 0.05). Different lower case letters superscripted indicate significant differences among SPI gels containing different concentrations of MCC (*p* < 0.05).

## Data Availability

Data is contained within the article.
